# Parallel VMamba and Attention-Based Pneumonia Severity Prediction from CXRs: A Robust Model with Segmented Lung Replacement Augmentation

**DOI:** 10.3390/diagnostics15111301

**Published:** 2025-05-22

**Authors:** Bouthaina Slika, Fadi Dornaika, Karim Hammoudi

**Affiliations:** 1 Department of Computer Science and Artificial Intelligence, University of the Basque Country UPV/EHU, 200018 San Sebastian, Spain; bslika001@ikasle.ehu.eus; 2 Faculty of Information Technology, Ho Chi Minh City Open University, Ho Chi Minh City 722000, Vietnam; 3 IKERBASQUE, Basque Foundation for Science, 48009 Bilbao, Spain; 4 Department of Computer Science, Institut de Recherche en Informatique, Mathématiques, Automatique et Signal, Université de Haute-Alsace, 68093 Mulhouse, France; karim.hammoudi@uha.fr

**Keywords:** pneumonia, lung diseases, automatic prediction, chest X-ray, severity quantification, mamba, data augmentation

## Abstract

**Background/Objectives:** Rapid and accurate assessment of lung diseases, like pneumonia, is critical for effective clinical decision-making, particularly during pandemics when disease progression can be severe. Early diagnosis plays a crucial role in preventing complications, necessitating the development of fast and efficient AI-based models for automated severity assessment. **Methods:** In this study, we introduce a novel approach that leverages VMamba, a state-of-the-art vision model based on the VisualStateSpace (VSS) framework and 2D-Selective-Scan (SS2D) spatial scanning, to enhance lung severity prediction. Integrated in a parallel multi-image regions approach, VMamba effectively captures global and local contextual features through structured state-space modeling, improving feature representation and robustness in medical image analysis. Additionally, we integrate a segmented lung replacement augmentation strategy to enhance data diversity and improve model generalization. The proposed method is trained on the RALO and COVID-19 datasets and compared against state-of-the-art models. **Results:** Experimental results demonstrate that our approach achieves superior performance, outperforming existing techniques in prediction accuracy and robustness. Key evaluation metrics, including Mean Absolute Error (MAE) and Pearson Correlation (PC), confirm the model’s effectiveness, while the incorporation of segmented lung replacement augmentation further enhances adaptability to diverse lung conditions. **Conclusions:** These findings highlight the potential of our method for reliable and immediate clinical applications in lung infection assessment.

## 1. Introduction

Pneumonia remains a significant global health concern, contributing to high morbidity and mortality rates, particularly among children, the elderly, and immunocompromised individuals [1]. Timely detection is essential to initiate treatment, prevent complications, and improve outcomes. Medical imaging plays a pivotal role in this process, with computed tomography (CT), chest X-ray (CXR), and lung ultrasound commonly used in clinical practice to support diagnosis, treatment planning, and disease monitoring. Among these modalities, CXRs are widely adopted due to their broad availability, cost-effectiveness, and reduced radiation exposure, making them the preferred first-line screening tool for pneumonia [2].

The widespread use of imaging has led to an overwhelming volume of data for radiologists, increasing the risk of diagnostic variability and interpretation fatigue. In response, artificial intelligence (AI) models have emerged as valuable tools to support automated disease analysis, offering the potential to enhance diagnostic consistency and alleviate clinician workload [3,4].

The development of deep learning has significantly transformed medical image analysis. By enabling automated feature extraction and complex pattern recognition from imaging data, deep learning methods have improved diagnostic precision and efficiency [5,6]. Unlike traditional machine learning approaches that rely on handcrafted features, deep learning methods—particularly convolutional neural networks (CNNs) and transformer-based architectures—can learn hierarchical representations directly from raw medical images. This capability allows deep learning models to excel in various medical imaging tasks, including disease detection, segmentation, severity quantification, and prognosis prediction [7,8]. When trained on large datasets, deep models can identify subtle radiographic signs that may be overlooked in manual reviews, contributing to more standardized and reproducible assessments [9,10,11].

CNNs have long been the foundation of medical image analysis, yet their limited ability to capture global dependencies has led to the adoption of alternative architectures [12]. Vision Transformers (ViTs), leveraging self-attention mechanisms, have demonstrated strong global feature learning but often require extensive data and computational resources [13]. Moreover, ViTs may struggle with capturing localized details critical to certain medical tasks. Recently, structured state-space models (SSMs), such as Mamba, have emerged as an effective alternative, combining memory-efficient sequential processing with scalability [14,15,16]. These models are particularly well-suited to tasks requiring a detailed understanding of anatomical structures and the spatial progression of disease.

In this study, we propose a deep learning framework that integrates a VMamba-based encoder with a parallel attention mechanism for estimating lung disease severity from CXR images. Unlike traditional classification models, our approach is tailored for regression-based severity scoring, enabling a more precise quantification of disease burden—an essential component for longitudinal patient monitoring and clinical decision support. A major challenge in severity assessment is the scarcity of labeled data, as scoring requires expert radiologists to assign structured annotations, making the process time-consuming and resource-intensive.

To address the challenge of limited severity-labeled datasets, we implement a tailored augmentation strategy that expands the training data while preserving critical clinical features. This approach enhances the model’s ability to recognize a broader range of severity levels, improving its generalization to unseen cases. By introducing diverse training samples that reflect varying disease patterns, our method strengthens the model’s capacity to differentiate severity stages with higher precision. Extensive experiments validate the effectiveness of this strategy, demonstrating that our model maintains strong predictive accuracy despite data constraints. These results underscore the potential of our framework in automating severity assessment, facilitating better patient monitoring, and streamlining clinical workflows.

The key contributions of this study are as follows:

- Development of an innovative deep learning framework that integrates a parallel model with a VMamba encoder, leveraging both channel and spatial attention mechanisms to enhance severity score prediction accuracy from CXRs.
- Introduction of a specialized augmentation strategy, focusing on segmented lung regions. This method incorporates self-replacement and cross-replacement techniques to increase dataset diversity.
- Comprehensive evaluations across multiple datasets, accompanied by ablation studies that assess the model’s generalizability and robustness under different conditions.

The remainder of this paper is structured as follows: Section 2 reviews existing literature and highlights recent advancements in pneumonia severity assessment. Section 3 introduces the proposed deep learning framework, detailing its architectural design and augmentation strategy for lung severity quantification. Section 4 outlines the evaluation methodology, including dataset descriptions, experimental results, and a thorough performance analysis. This section also presents findings from ablation studies, offering insights into the impact of various model components. Lastly, Section 5 summarizes the key contributions of this work and provides concluding remarks.

## 2. Related Work

Deep learning has revolutionized medical image analysis by enabling automated feature extraction and pattern recognition, significantly improving diagnostic accuracy and efficiency [3]. CNN architectures such as ResNet, DenseNet, and EfficientNet have demonstrated state-of-the-art performance in detecting lung conditions like pneumonia and tuberculosis, proving their effectiveness in medical imaging [17,18,19]. More recently, transformer-based models have introduced self-attention mechanisms to enhance feature representation and capture global contextual information, addressing some of CNNs’ limitations [20]. Additionally, hybrid approaches that integrate CNNs with attention mechanisms have been developed to improve both analysis and localization of pathological regions [21]. These advancements have shifted the focus from basic disease classification to severity quantification, making AI-driven diagnostic tools more applicable to clinical practice.

Deep learning has been extensively utilized for severity quantification across various medical imaging modalities, employing regression-based estimation to provide continuous severity scores rather than discrete classifications. In brain MRI analysis, deep regression networks have been used to assess stroke severity, while in fundus imaging, they have facilitated automatic grading of diabetic retinopathy [22,23]. Similarly, deep learning techniques have been applied to quantify the severity of pneumonia, COVID-19, and chronic obstructive pulmonary disease (COPD) [24]. CNN-based architectures, including 3D U-Net and DenseNet, have been particularly effective in extracting spatial and textural features from CT scans, enabling accurate estimation of lung opacity and lesion extent [25,26].

Several deep learning approaches have been proposed for CXR-based severity quantification, aiming to automate lung abnormality scoring based on radiological criteria [27,28]. Wong et al. [29] utilized a ResNet-based regression model trained on expert-annotated severity scores to estimate lung involvement in CXR images. Tang et al. [30] developed a CNN-driven framework for COVID-19 severity assessment, while Wehbe et al. [31] employed a multi-task learning strategy using DenseNet-121 to simultaneously detect COVID-19 and predict severity levels. Transformer-based models have also shown promise in this domain [32,33], with Zhang et al. [34] leveraging Vision Transformers (ViTs) for pneumonia severity estimation. Additionally, hybrid architectures, including the Swin Transformer, have demonstrated improved spatial feature weighting, enhancing interpretability and regression accuracy [35,36,37]. The adoption of regression-based deep learning models has facilitated more precise and standardized severity scoring, contributing to improved clinical decision-making [38,39].

Recently, Mamba-based architectures have emerged as a powerful alternative for modeling long-range reliances with linear computational complexity, making them particularly effective for medical imaging tasks [15,16,40,41,42]. Unlike transformers, which suffer from quadratic complexity, Mamba efficiently handles sequential dependencies, enhancing performance across classification, segmentation, and detection applications [43,44,45,46,47]. Several Mamba-based models have been developed to address different challenges in medical imaging. The Tri-Plane Mamba model has been adapted for enhanced 3D medical image segmentation [48]. Additionally, MedMamba [49] integrates convolutional layers with state-space models to boost classification accuracy, while architectures like Swin-UMamba [50] and Mamba-UNet [51] employ Mamba’s computational efficiency within UNet frameworks, yielding superior segmentation results. Hybrid models, such as VM-UNet [52] and Weak-Mamba-UNet [53], further refine segmentation quality while maintaining efficiency, demonstrating the growing potential of Mamba-based architectures in medical image analysis.

Although Mamba-based models have primarily been utilized for segmentation tasks in CXR analysis—such as identifying lung boundaries, detecting pathological regions, or extracting anatomical structures—our study adopts a different perspective. Rather than focusing on segmentation, we leverage Mamba for a regression-based approach, predicting continuous severity scores directly from CXR images. This strategy facilitates a more precise assessment of disease progression, providing valuable insights to support clinical decision-making and patient management.

## 3. Proposed Methodology

### 3.1. Proposed Model

**Figure 1:**
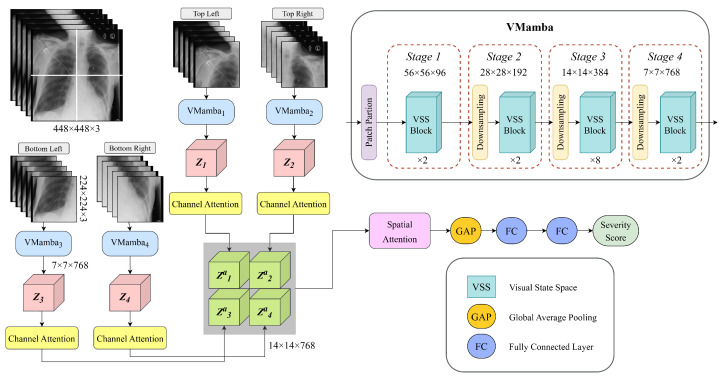
Illustration of the proposed model. The structure of VMamba and the legend are embedded in the figure.

Evaluating lung disease severity from CXR images necessitates a model capable of capturing both localized anatomical features and broader pathological patterns. The proposed approach is designed to predict a quantitative severity score from a given CXR image. It employs a complex architecture combining parallel Mamba-based encoders for feature extraction with sequence modeling, enhanced by channel and spatial attention mechanisms. This structured design acts on multiple sub-images and progressively refines representations across these different regions. The architecture’s detailed structure, along with illustrative blocks, is depicted in Figure 1. The model is trained in an end-to-end fashion.

The processing pipeline begins with the raw CXR image, denoted as *I*, which is partitioned into four regions to facilitate localized feature extraction. To ensure uniform processing, the original image dimensions are resized to 2W×2H×C, resulting in each quadrant having a final size of W×H×C. These quadrants are processed in parallel, with each sub-image undergoing a series of transformation layers for deep feature extraction.

At each stage of the pipeline, images are passed through VMamba-based processing units, leveraging the recently proposed VMamba model by Liu et al. [54]. The process begins with patch partitioning in each line of the parallel encoder. The input image is divided into a series of patches, where each patch has size *P* × *P* and $N=\frac{H\times W}{P^{2}}$ is the number of image patches to ensure structured and efficient processing. This step enables the model to handle localized regions of the image while maintaining the overall spatial relationships.

**Figure 2:**
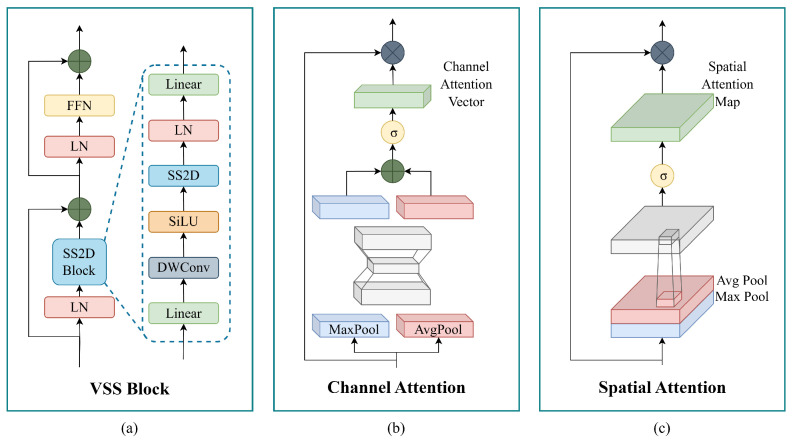
Illustration of the structure of the VSS Block (**a**), Channel Attention (**b**), and Spatial Attention (**c**).

Following partitioning, the image enters a series of four stages (k=1,2,3,4), where the image patches are processed. In each stage, the patches are input into layers of Vision State Space (VSS) Blocks, a core component of VMamba. It is designed for efficient spatial feature extraction. As shown in Figure 2a, the block consists of two primary processing branches. The first branch features an SS2D Block, which applies state-space modeling in two dimensions (2D) to effectively capture long-range dependencies within the image. SS2D processes input patches through three steps: cross-scan, selective scanning with S6 blocks, and cross-merge, where patches are unfolded into sequences along multiple traversal paths and processed in parallel. This approach allows each pixel to integrate information from all others across different directions, creating global receptive fields in the 2D space [54]. This is followed by Layer Normalization (LN) to stabilize the feature distribution before merging with the second branch. The second branch includes a depthwise convolution (DWConv) layer, followed by a SiLU activation function to introduce non-linearity. Next, a series of linear transformations and normalization layers further refine the extracted features. Finally, a feed-forward network (FFN) enhances feature representation, with residual connections ensuring stable gradient flow.

After patch partitioning, at the first stage, two VSS Blocks are applied to extract low-level spatial features, resulting in a feature map. In the following stages, a downsampling operation is performed, followed by $L_{k}$ layers of VSS Blocks. The downsampling effectively decreases the spatial dimensions while increasing feature depth. The resolution of the input tensor and the number of channels at each stage are progressively adjusted following the following equation:

(1) $$\frac{H}{2^{k+1}}\times \frac{W}{2^{k+1}}\times C_{k},$$

where *H* is the image height, *W* is the image width, $C_{k}$ is the number of channels after the $k^{\mathrm{th}}$ stage and is calculated as $C_{k}=C\cdot 2^{k+4}$. By progressively refining the image representation through hierarchical stages, this VMamba-based encoder efficiently balances local and global feature extraction. The final output of each VMamba typically has a small spatial resolution with a high number of channels, providing a rich representation that combines global context with fine-grained details. In our setup, H=224, W=224, P=4, C=3 and $L_{k}=\left\{2,2,8,2\right\}$.

Each VMamba unit produces an embedding $Z_{i}$ at Stage 4, encapsulating the extracted feature information. A channel attention mechanism is then applied to refine these features by emphasizing the most informative channels. This mechanism calculates importance scores using global pooling and nonlinear transformations, generating a 1D attention vector (1×1×768), which re-weights the feature maps to prioritize significant details for severity prediction. The attention vector is computed as:

(2) $$M_{i}^{CH}=\sigma \left[MLP\left(\mathrm{AP}\left(Z_{i}\right)\right)+MLP\left(\mathrm{MP}\left(Z_{i}\right)\right)\right]$$

where $M_{i}^{CH}$ represents the computed channel attention scores of the $i^{\mathrm{th}}$ encoder, $Z_{i}$ is the input feature, AP is average pooling, MP is max pooling, σ is the sigmoid activation, and MLP refers to a multi-layer perceptron. The final feature representation is obtained through channel-wise scaling:

(3) $$Z_{i}^{a}=M_{i}^{CH}⊙Z_{i}$$

where $Z_{i}^{a}$ is the refined output of the $i^{\mathrm{th}}$ pipeline. The illustration of the channel attention module is represented in Figure 2b. To reconstruct spatial relationships, the processed tensors from different sub-images are combined along horizontal and vertical dimensions, aligning them to restore their original spatial structure within the image *I*, as illustrated in Figure 1.

After aggregation, the feature tensor $Z^{a}$ undergoes spatial attention processing, which selectively amplifies critical spatial regions while suppressing less significant areas. This mechanism directs the model’s focus toward the most informative lung regions, particularly those exhibiting pathological patterns relevant to severity estimation. The spatial attention module achieves this by first extracting essential spatial information through average pooling and max pooling operations across the channel dimension. The pooled feature maps are then concatenated to form a compact spatial descriptor, which is used to generate an attention map:

(4) $$M^{S}=\sigma \left[conv^{7\times 7}\left(\mathrm{AP}\left(Z^{a}\right);\mathrm{MP}\left(Z^{a}\right)\right)\right]$$

where $M^{S}$ represents the computed spatial attention map, $Z^{a}$ is the input tensor, σ is the sigmoid activation function, and $conv^{7\times 7}$ is a convolutional layer with a 7×7 kernel. To refine the feature representation, the spatial attention map is applied to the input tensor through element-wise multiplication, denoted as ⊗:

(5) $$S=M^{S}⊗Z^{a}$$

where *S* is the final output, in which the original feature map $Z^{a}$ is re-weighted according to the computed spatial attention values. This step ensures that the model prioritizes the most relevant spatial features, enhancing its ability to assess severity. The illustration of the spatial attention is represented in Figure 2c.

After applying spatial attention, the refined feature representation tensor *S* undergoes a Global Average Pooling (GAP) operation, which condenses the spatial dimensions by computing the mean activation for each feature channel. This transformation generates a compact feature vector of size equal to the number of channels (784). This pooled feature vector is then passed through a series of Fully Connected (FC) layers, which learn high-level abstractions and relationships within the feature space. The final predicted scalar represents the quantitative severity score of the lung disease.

By integrating hierarchical VMamba-based features with attention mechanisms, this architecture effectively captures both local anatomical structures and broader pathological patterns. The combination of feature extraction, attention-based refinement, and fully connected transformations ensures robust severity assessment tailored for complex medical imaging tasks.

### 3.2. Data Augmentation: Segmented Lung Replacement

**Figure 3:**
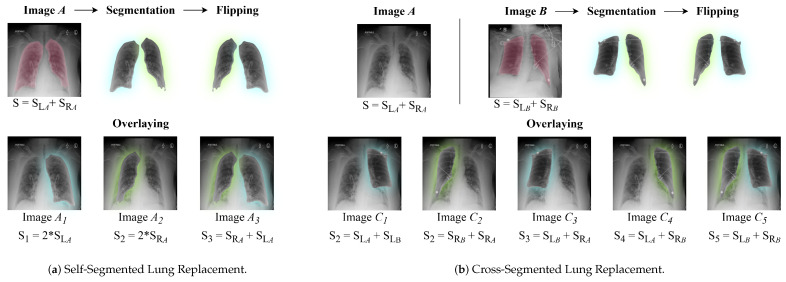
Our Proposed Lung Augmentation Techniques. The ground-truth (S) scores are calculated along with the produced images.

Severity-labeled CXR datasets are inherently limited due to the need for expert radiologist annotations, which are both costly and time-consuming to produce. As a result, the available training data often lack sufficient diversity and may exhibit imbalanced distributions of severity scores. To address this, we introduce a novel segmentation-guided augmentation strategy called Segmented Lung Replacement (SLR) Augmentation. This technique generates a diverse set of augmented images through the replacement of lung regions within or across patient images. This approach synthetically increases the dataset size while preserving anatomical and clinical validity. This enhanced diversity improves the model’s exposure to a broader spectrum of pathological patterns and leads to more robust severity prediction, as confirmed by our performance results. Our augmentation framework is systematically categorized into two distinct types, each designed to introduce meaningful variations while maintaining diagnostic relevance. These categories, along with their implementation details, are elaborated below and visually depicted in Figure 3.

#### 3.2.1. Self-Segmented Lung Replacement

The Self-Segmented Lung Replacement (Self-SLR) augmentation technique generates diverse synthetic samples by segmenting and selectively modifying specific lung regions within a given input image. The process begins with the precise segmentation of the lungs using a pre-trained UNet++ model [55], which isolates the left and right lung regions from the original CXR image. Once segmented, each lung region is transformed by applying a horizontal flip operation, generating a mirrored version of the lung. These modified lung regions are then seamlessly reintegrated into the original CXR, ensuring anatomical consistency and plausibility. To introduce greater diversity while maintaining clinical relevance, we apply three distinct variations of this augmentation: (1) replacing only the right lung, (2) replacing only the left lung, and (3) replacing both lungs simultaneously. The severity score of each augmented image is determined by summing the ground-truth severity scores of the replaced lung regions. This ensures that the severity labels remain consistent with the introduced modifications. A step-by-step visual description of the augmentation process is provided in Figure 3a. By selectively modifying lung regions rather than altering the entire image, this method preserves the underlying diagnostic structures while significantly enhancing dataset variability. This strategy improves the model’s ability to generalize across diverse anatomical variations, ultimately strengthening its robustness in severity prediction tasks.

#### 3.2.2. Cross-Segmented Lung Replacement

Cross-Segmented Lung Replacement (Cross-SLR) method enhances dataset diversity by integrating segmented lung regions from multiple input images. Instead of modifying lung regions within the same image, this approach extracts lung segments from two or more distinct CXR images and swaps corresponding regions while ensuring proper anatomical alignment. By replacing a lung from one patient’s X-ray with the corresponding lung from another, this technique generates composite images that better capture inter-patient variability. Similar to the Self-SLR technique, the process begins with precise segmentation of lung regions using UNet++ [55]. Once segmented, the extracted lungs are utilized to replace lung regions in other images based on predefined augmentation modes. This strategy enables five different composite image configurations, where lung swapping can be applied to either a single lung—with or without flipping—or to both lungs simultaneously. The augmentation process is detailed and visually represented in Figure 3b. The severity score for each augmented image is determined by summing the ground-truth severity scores of the replaced lung regions, ensuring that the augmented dataset maintains meaningful clinical annotations. By leveraging segmentation outputs across different patients, this method significantly increases the number of unique training samples. The ability to combine lung regions from different images introduces greater structural and pathological diversity, exposing the model to a broader spectrum of severity patterns. This augmentation ultimately improves the model’s generalization capability, making it more robust in real-world medical imaging scenarios.

In our study, we employed both augmentation techniques as online strategies to mitigate the limitations of medical imaging datasets. These methods increase training samples and introduce diverse variations, enhancing model robustness and generalization. By dynamically generating augmented images during training, the model continuously encounters new variations, reducing overfitting risk.

Each epoch applies Self-SLR to produce three additional images and Cross-SLR to generate five, expanding a batch from 16 to 144 images—a 9× increase. Given an initial dataset of 5634 images, this results in 50,706 images per epoch. As a regression task, augmentations retain corresponding severity scores for learning consistency, as shown in Figure 3a,b.

This framework is particularly suited for datasets with localized or global severity scores, making it effective for regression-based medical imaging. By maintaining anatomical accuracy and introducing clinically relevant variations, our approach enhances performance in lung disease severity prediction.

## 4. Performance Evaluation

### 4.1. Datasets

This study aims to explore the effectiveness of deep learning models in predicting the severity of lung diseases using CXR images. By using CXR datasets that are annotated with quantitative severity scores, we provide a detailed assessment of the severity of lung infections. These datasets are used to train and validate our model, ensuring that it can precisely quantify disease progression by analyzing key features within the radiological images. We used two datasets to test our model using CXRs. The RALO dataset and the COVID-19 dataset. Table 1 and Table 2 show the main characteristics of these datasets and their associated scores respectively.

#### 4.1.1. RALO Dataset

The Radiographic Assessment of Lung Opacity (RALO) Score dataset comprises 2373 CXR images, initially introduced by Cohen et al. [58]. These images were assessed by two experienced radiologists from Stony Brook Medicine, who assigned severity scores to each image based on the lung infection level. The dataset was previously split into a training set of 1878 images and a test set of 495 images [56].

The severity assessment in this dataset is based on two primary radiological factors: Geographic Extent (GE) and Lung Opacity (LO). GE evaluates the extent of lung involvement by ground-glass opacity or consolidation, with separate scores for the left and right lungs. Each lung is assigned a score between 0 (no consolidation) and 4 (extensive consolidation), and the total GE score is calculated by summing the individual scores for both lungs. On the other hand, LO measures the intensity of lung opacity, with scores ranging from 0 (no opacity) to 4 (complete whiteout) per lung. The combined LO score can range from 0 to 8, depending on the sum of the individual lung opacity ratings.

To ensure accurate ground-truth labels, the final severity score is determined by averaging the assessments of two radiologists, with scores ranging from 0 to 8 expressed in discrete intervals with 0.5 as an increment. To further enhance the model’s training diversity, an offline augmentation technique—based on the replacement of lung regions and their corresponding severity scores—was applied, following our previous approach [59]. This augmentation process expanded the training dataset to 5634 images, while the test set remained at 495 images. This strategy introduces increased variability in the dataset, enabling the model to better generalize across a wide range of severity levels.

#### 4.1.2. COVID-19 Dataset

In this study, we also utilized a dataset compiled by Danilov et al. [57]. It contains 1364 CXR images from patients with COVID-19 and healthy individuals with no visible lung abnormalities. The dataset is balanced, with 580 images (43%) from COVID-19-positive patients and 784 images (57%) from healthy controls, making it suitable for both training and evaluation purposes. Each image in the dataset is annotated with a COVID-19 score. It is a severity score ranging from 0 to 6 with an increment of 1, indicating the level of lung involvement. A score of 0 denotes no abnormalities, while a score of 6 indicates severe pathology, with more than 85% of the lung area affected. Intermediate scores represent varying degrees of mild to extensive lung damage, offering a detailed severity scale.

This dataset is crucial for training deep learning models focused on COVID-19 severity assessment. By incorporating both healthy and infected lung images, it enables models to differentiate between normal and abnormal lung conditions, thereby enhancing their diagnostic performance and reliability in clinical settings.

### 4.2. Experimental Results and Comparison

In this study, we present a structured deep learning framework for predicting lung disease severity from CXR images using a regression-based approach. The primary dataset, RALO, comprises 5634 CXR images, each labeled with GE and LO scores on a scale from 0 to 8, representing varying degrees of lung involvement from mild to severe. Our methodology encompasses data processing, splitting, and deep learning-driven severity estimation to ensure precise and reliable quantification.

The data processing pipeline includes resizing and normalization to ensure a consistent input shape and intensity scaling, which are required for the patch-based VMamba encoder. These steps help standardize the input and stabilize model convergence. In addition, lung segmentation is used to extract anatomically relevant lung regions that guide our SLR augmentation strategy. By isolating lung fields, we enable the application of our proposed augmentations, which generate diverse yet clinically valid training samples. Both Self-SLR and Cross-SLR are applied dynamically during training as online augmentation strategies, ensuring that each batch contains diverse augmented samples, thereby enhancing model generalization and robustness to variations in lung pathology.

**Figure 4:**
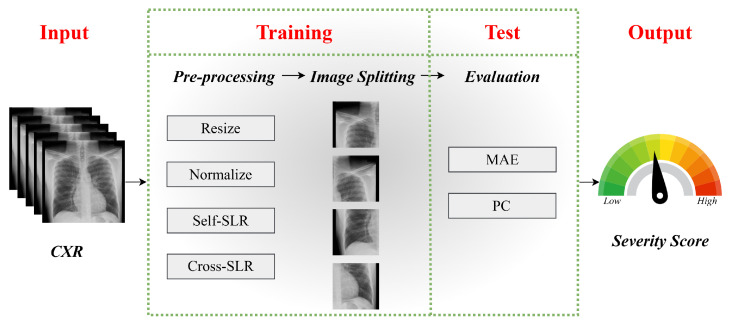
The workflow of score prediction from CXR.

Following augmentation, an image-splitting technique is implemented, dividing each CXR into four quadrants. This enables the model to focus on localized lung regions, improving its ability to detect fine-grained pathological patterns that contribute to severity assessment. The processed images are then fed into the proposed deep learning model, which predicts a continuous severity score indicative of disease progression. The final output provides a quantitative severity score, aiding clinicians in evaluating disease progression and supporting informed medical decision-making. Figure 4 visually outlines the entire workflow, illustrating the sequential steps of our proposed work.

In this study, we utilized the $L_{1}$ loss function for severity score regression to enhance robustness against outliers. The AdamW optimizer, with a learning rate of $10^{-5}$, was chosen to minimize weight decay and improve generalization. To optimize training dynamics, we applied a cosine annealing warm restarts scheduler, with the initial restart period matching the training loader length and subsequent periods doubling progressively. This approach helped the model escape local minima and achieve better convergence. All experiments were conducted on an NVIDIA Titan X GPU with CUDA 12.0, ensuring efficient processing of large-scale CXR datasets.

To evaluate our model’s effectiveness, we used Mean Absolute Error (MAE) and Pearson’s correlation coefficient (PC) to assess agreement with radiologist-assigned severity scores. MAE quantifies prediction accuracy, with lower values indicating better performance, while PC measures the strength of the linear relationship, ranging from −1 (negative correlation) to +1 (perfect correlation). Higher PC values and lower MAE confirm the model’s reliability in severity quantification.

To comprehensively assess the effectiveness of our proposed model, we performed a comparative analysis against multiple state-of-the-art deep learning architectures used for severity assessment. This evaluation aimed to demonstrate the advantages of our approach in accurately predicting lung disease severity through quantitative analysis.

The benchmark models selected for comparison include both convolutional neural networks (CNNs) and transformer-based architectures. Specifically, the models evaluated in this study are COVID-NET [29], COVID-NET S [60], ResNet50 [61], Swin Transformer [62], XceptionNet [63], Feature Extraction Model [56], MobileNetV3 [64], InceptionNet [65], ViTReg-IP [59], and MViTReg-IP [66]. To ensure a fair and unbiased comparison, all models were trained under identical conditions, utilizing the same dataset and evaluation metrics.

The findings of this comparative analysis are summarized in Table 3, where our proposed model outperforms existing architectures by exhibiting lower error rates and stronger correlation with the baseline severity scores. The number of parameters is also reported in the table to provide information about the models’ complexity.

To further assess the generalizability of our proposed model, we conducted additional experiments using the COVID-19 dataset by Danilov et al. [57]. This dataset contains CXR images annotated with severity scores ranging from 0 to 6, reflecting the extent of lung involvement in COVID-19 cases. Unlike the RALO dataset, which provides per-lung severity annotations, this dataset assigns a single global severity score per image, presenting a distinct challenge for model evaluation. The lack of individual lung scores prevented us from directly applying our SLR techniques, which rely on manipulating specific lung regions. To address this, we adapted both Self-SLR and Cross-SLR at the full lung level, ensuring that the augmentation process remained beneficial in enhancing model robustness. Although there is a lack of per-lung severity scores, this evaluation provided key insights into the model’s adaptability. Table 4 presents the results of training our model on the COVID-19 dataset.

In our ablation studies, we investigated the influence of the proposed data augmentation techniques on the performance of our model. This experiment aimed to determine how the two augmentation strategies improve the model’s ability to detect complex lung features in CXR images. We evaluated four configurations: training without any augmentation, applying traditional augmentations including horizontal flipping, ±10° rotation, and Gaussian noise injection, applying only Self-SLR, and using only Cross-SLR. The baseline condition, without augmentation, allowed us to assess the model’s raw performance without any data adjustments. Both augmentation methods introduce anatomical variations while preserving the consistency of the original patient data, thus enhancing the diversity of the training set by incorporating variations from different patients. The results are presented in Table 5.

To investigate the effect of architectural design choices in our model, we performed an ablation study focusing on the role of attention mechanisms. The results, presented in Table 6, explore the impact of both channel and spatial attention on the accuracy of lung severity score predictions. We examined four configurations: without any attention layers, with spatial attention, with channel attention, and with both attentions. The configuration without attention layers serves as the baseline to evaluate the model’s performance in the absence of any attention-enhanced features. By incorporating attention mechanisms, we aimed to determine whether considering spatial and channel dependencies contributes to performance improvement.

### 4.3. Discussion

Table 3 showcases the exceptional performance of our proposed model in assessing lung disease severity using the RALO CXR dataset. Our model achieved the lowest MAE values of 0.356 for GE and 0.338 for LO, along with the highest PC scores of 0.970 for GE and 0.941 for LO. These results demonstrate a strong agreement between the model’s predictions and the ground truth severity scores, underscoring its accuracy and reliability in severity quantification. The significant performance advantage over competing models further highlights the effectiveness of our approach. The proposed model contains approximately 86 M parameters. While some models, such as MobileNetV3, offer lightweight deployment with few parameters (4.5 M), their prediction accuracy is notably lower. Conversely, our model achieves top performance although it have a high parameter count. It is worth noting that once training is complete, the model performs score prediction automatically and within milliseconds of image input. This enables immediate, post-imaging severity scoring without the need for radiologist intervention, making it highly suitable for time-sensitive clinical environments.

The results in Table 4 highlight the excellent generalization capability of our proposed model across various scoring systems and datasets. When trained on the COVID-19 dataset, the model achieves strong predictive accuracy, with an MAE of 0.322 and a PC of 0.921, despite differences in dataset size and scoring range. When compared to the RALO dataset, the model performs consistently well on the COVID-19 dataset, demonstrating its robustness in capturing variations in disease severity. This consistent performance across datasets with differing score distributions underscores the adaptability of our model and its potential for clinical use in lung severity assessment.

The superior performance and low MAE of the proposed model result from a carefully integrated architecture that combines VMamba’s structured state-space modeling for capturing long-range spatial dependencies, a parallel dual-branch design that mimics clinical assessment by processing left and right lungs independently, and hybrid spatial and channel attention mechanisms that guide the model to focus on the most relevant anatomical regions and feature channels. Together, these components enable precise, context-aware severity estimation from CXRs, outperforming traditional CNNs and Transformers in both accuracy and clinical relevance.

Additionally, the proposed model achieves an average inference time of a few milliseconds per image, which supports integration into real-time clinical workflows. Beyond speed, its ability to automatically generate quantitative severity scores without the need for radiologist input at test time offers significant utility in healthcare. Patients or clinicians can obtain immediate feedback following image acquisition, enabling timely decision-making and potentially increasing access to care in under-resourced regions. Moreover, the low MAE values, validated against expert radiologist annotations, demonstrate the model’s ability to deliver clinically meaningful and trustworthy severity predictions.

Table 5 highlights the significant improvements our augmentation strategies bring to model performance. While the baseline model, trained without any augmentation, shows competitive results, the incorporation of augmentation techniques leads to marked improvements. The Self-SLR method, which alters lung regions within the same image, enhances generalization, improving both MAE and PC for GE and LO scores. The Cross-SLR method shows even better results by diversifying the training set, allowing the model to capture more complex lung features. This approach achieves the lowest MAE (0.360 for GE, 0.342 for LO) and the highest PC (0.966 for GE, 0.938 for LO). CrossSLR introduces a broader spectrum of training diversity by recombining segmented lungs from different patients with varying severity profiles. This approach generates synthetic but anatomically valid CXR images that are not found in the original dataset. As a result, the model is exposed to a wider distribution of severity combinations, such as pairing a severely affected lung with a healthy one, which enhances its ability to generalize to unseen test cases. In contrast, SelfSLR performs augmentation within the same patient image, preserving local coherence but offering a more limited variability. This difference in augmentation diversity explains the performance improvement of CrossSLR over SelfSLR, as shown in Table 5.

To better assess the advantage of the proposed augmentation methods, we conducted an additional baseline experiment using traditional augmentation techniques. This included horizontal flipping, ±10° rotation, and Gaussian noise injection, which are common practices in medical image preprocessing. As shown in Table 5, while these methods provide a minor improvement over training without augmentation (MAE 0.372 for GE and 0.353 for LO), they are still outperformed by both Self-SLR and Cross-SLR techniques.

The results presented in Table 6 underscore the significant impact of attention mechanisms on the model’s performance. The baseline model, which does not incorporate any attention mechanisms, demonstrates the poorest performance, with the highest MAE (0.432 for GE, 0.402 for LO) and the lowest PC (0.939 for GE, 0.922 for LO). These results indicate that the model struggles to effectively capture the necessary spatial and contextual information required for accurate severity prediction. Introducing spatial attention alone leads to a notable improvement, as the model can now better focus on relevant regions within the image. This results in a decrease in MAE and an increase in PC, demonstrating the positive effect of spatial attention in guiding the model’s focus toward critical lung features. Similarly, incorporating channel attention independently also results in improved performance. Channel attention facilitates better interaction between different feature representations, allowing the model to capture more complex lung patterns. This enhancement is reflected in the reduction of MAE and the increase in PC. However, the most substantial improvement occurs when both spatial and channel attention mechanisms are employed simultaneously. In this configuration, the model achieves the best performance, with the lowest MAE (0.356 for GE, 0.338 for LO) and the highest PC values (0.970 for GE, 0.941 for LO). These results demonstrate that spatial attention and channel attention work synergistically to refine the model’s ability to focus on critical lung features while maintaining contextual awareness across the image.

## 5. Conclusions

Pneumonia is a critical respiratory condition that requires accurate and timely detection for effective treatment and prevention of its spread. In this work, we propose a novel deep learning framework that leverages a parallel VMamba architecture, integrating VSS-based encoding along with channel and spatial attention mechanisms to improve pneumonia severity assessment from CXR images. This architecture allows for a more nuanced and interpretable analysis of lung disease severity, offering better insights for clinicians.

Our method’s superior performance is evidenced by its significantly lower MAE and higher PC values compared to CNN and Transformer baselines. These gains are further enhanced by our anatomically consistent augmentation, demonstrating the value of domain-specific data manipulation. Unlike prior works that rely on classification tasks, we provide a regression-based solution designed for severity prediction. By generating more precise and quantitative severity estimates, the model assists clinicians in making informed diagnostic and treatment decisions. Furthermore, our ablation studies demonstrate the importance of the novel augmentation strategies used in the framework, which contribute significantly to enhancing model generalization and robustness.

While this study evaluates the performance of our framework on the RALO and COVID-19 datasets, further studies are required to validate the proposed model across diverse datasets and clinical environments to ensure its generalizability and robustness. Future work will focus on testing the model on larger, more heterogeneous datasets and comparing its performance to existing state-of-the-art approaches to establish its clinical applicability and reliability.

We acknowledge the valuable contributions of expert radiologists whose annotations were essential for validating our model’s accuracy. Future work will involve further clinical validation in collaboration with medical professionals to assess real-world applicability. One limitation of this study is that pleural effusion, though clinically important, is not explicitly annotated in the RALO or COVID-19 datasets. While the model may implicitly associate pleural effusion patterns with higher severity scores, especially when effusion co-occurs with consolidation, the absence of dedicated labels restricts the interpretability of such features. Future work should incorporate datasets with multi-label annotations to enable the model to distinguish between pleural effusion, lung consolidation, and other thoracic findings, thereby enhancing its diagnostic utility and alignment with clinical reality. Another limitation of our approach is the need for individual lung scoring to ensure that our proposed augmentation methods are applied completely and accurately, which may require additional manual annotation or refinement in certain clinical scenarios. Ultimately, our approach offers the potential for more accurate, efficient, and automated pneumonia severity estimation, enhancing diagnostic accuracy and supporting more effective clinical decision-making.

## Figures and Tables

**Table 1:** Details of the Datasets Used in our Work.

	Data			
Dataset	Size	Training	Validation	Test
RALO [56]	224 × 224 × 3	5634	-	495
COVID-19 [57]	224 × 224 × 3	1090	135	139

**Table 2:** Details of the Annotations of the Images Used in our Work.

	Score						
Dataset	Type	Min	Max	Increment	Mean	Std	Variance
RALO [56]	GE	0	8.0	0.5	5.1075	2.0915	4.3745
	LO	0	8.0	0.5	3.7355	1.5137	2.2913
COVID-19 [57]	COVID-19 score	0	6.0	1	1.6015	2.1908	4.7998

**Table 3:** Performance Evaluation of the Proposed Method vs. State-of-the-Art Techniques on the RALO CXR Dataset.

	GE		LO		
Model	MAE ↓	PC ↑	MAE ↓	PC ↑	Nb of Pram.
COVID-NET [29]	4.458	0.549	2.242	0.535	12 M
COVID-NET-S [60]	4.698	0.591	2.254	0.529	12 M
ResNet50 [61]	1.094	0.688	1.061	0.431	23 M
Swin Transformer [62]	0.916	0.817	0.803	0.697	29 M
XceptionNet [63]	0.854	0.821	0.768	0.701	23 M
Feature Extraction [56]	0.967	0.753	0.865	0.711	20 M
MobileNetV3 [64]	0.847	0.827	0.732	0.738	4.2 M
InceptionNet [65]	0.702	0.886	0.609	0.829	24 M
ViTReg-IP [59]	0.565	0.925	0.510	0.857	5.5 M
MViTReg-IP [66]	0.531	0.938	0.462	0.881	11.2 M
Ours	0.356	0.970	0.338	0.941	86 M

**Table 4:** The Performance of Our Model when Trained on the RALO and COVID-19 Datasets.

Dataset	Score	Train Size	Test Size	MAE ↓	PC ↑
RALO [58]	Geographic Extent	5634	495	0.356	0.970
RALO [58]	Lung Opacity	5634	495	0.338	0.941
COVID-19 [57]	COVID Score	1090	139	0.322	0.921

**Table 5:** Performance Evaluation of Our Proposed Data Augmentation Methods in Our Model.

	GE		LO	
Augmentation	MAE ↓	PC ↑	MAE ↓	PC ↑
No Augmentation	0.374	0.958	0.354	0.927
Traditional Augmentation	0.372	0.959	0.353	0.927
Self-SLR	0.366	0.963	0.348	0.934
Cross-SLR	0.360	0.966	0.342	0.938

**Table 6:** Performance Evaluation of Attention Layers in Our Model.

Attention		GE		LO	
Channel Attention	Spacial Attention	MAE ↓	PC ↑	MAE ↓	PC ↑
×	×	0.432	0.939	0.402	0.922
×	✔	0.367	0.962	0.349	0.930
✔	×	0.362	0.966	0.341	0.937
✔	✔	0.356	0.970	0.338	0.941

## Data Availability

The data used in this study are mentioned in Section 4.1.
